# Valorization of Dairy and Plant By-Products as Functional Ingredients in Kurt (Dried Fermented Milk Product): Effects on Nutritional, Physicochemical, and Sensory Properties

**DOI:** 10.3390/foods15020369

**Published:** 2026-01-20

**Authors:** Zhanar Kalibekkyzy, Shugyla Zhakupbekova, Maksim Rebezov, Almagul Nurgazezova, Gulnur Nurymkhan, Samat Kassymov, Sholpan Baytukenova, Akmaral Mateyeva, Aigul Maizhanova, Zarina Kapshakbayeva

**Affiliations:** 1 Department of Food Technology, Shakarim University, 20A Glinki Street, Semey 071412, Kazakhstan; zhanar_moldabaeva@mail.ru (Z.K.); almanya1975@mail.ru (A.N.); samat-kasymov@mail.ru (S.K.); fquekm2710@mail.ru (A.M.); 2 Department of Scientific Research, Gorbatov Research Center for Food Systems, 26 Talalikhin St., Moscow 109316, Russia; rebezov@ya.ru; 3 Faculty of Biotechnology and Food Engineering, Ural State Agrarian University, 42 Karl Liebknecht St., Yekaterinburg 620075, Russia; 4 Department of Technology of Food and Processing Industries, S. Seifullin Kazakh Agrotechnical Research University, Zhenis Avenue 62, Astana 010011, Kazakhstan; baytukenovasholpan@gmail.com; 5 BAYEL-KZ LLP, 70/27 Kulager Microdistrict, Almaty 050000, Kazakhstan; akmaral79@list.ru; 6 Department of Biotechnology, Toraygyrov University, 64 Lomova Street, Pavlodar 140000, Kazakhstan; z.k.87@mail.ru

**Keywords:** kurt, functional dairy product, whey ingredients, buttermilk protein sediment, sensory evaluation, mineral enrichment, amino acid profile

## Abstract

This study developed enriched kurt formulations using buttermilk protein sediment, spray-dried whey, soy protein concentrate, and flaxseed cake, and assessed their effects on composition, physicochemical parameters, microbiological stability, and sensory quality. Protein content increased from 46.2% in the control to 48.7–52.4% in experimental samples. Calcium levels rose from 750 mg/100 g to 856 mg/100 g in Experiment 1 and 880.7 mg/100 g in Experiment 3 (*p* < 0.05), demonstrating strong mineral enhancement. Moisture decreased from 13.61% in the control to 11.68–12.90% in enriched variants (*p* < 0.05), indicating more efficient dehydration and a denser structure. pH remained within 4.1–4.3 and water activity stayed below 0.60, supporting long-term microbial stability. Amino acid profiling showed higher levels of essential amino acids, particularly leucine and lysine, in samples containing buttermilk protein sediment and whey. Microbiological analysis confirmed low total viable counts values (9.0 × 10^2^–1.2 × 10^3^ CFU/g), consistent with the high acidity and low moisture of traditional kurt. Sensory evaluation revealed significant variation among formulations. The control and Experiment 2 received the highest taste and aroma scores (4.67 points), while Experiment 3 showed the lowest values (3.33 points; *p* < 0.05). Appearance scores decreased notably in darker samples, with Experiment 3 showing a reduction from 4.67 to 2.67 points (*p* < 0.05). Texture also differed across variants; Experiment 2 maintained acceptable hardness and cohesiveness (4.33 points), whereas Experiment 3 displayed increased crumbliness (3.0 points; *p* < 0.05). The findings demonstrate that functional enrichment of kurt is feasible when ingredient levels remain within an optimal range. The Experiment 2 formulation achieved improved nutritional value without compromising sensory quality, providing a promising basis for further technological development and commercial application.

## 1. Introduction

Fermented dairy products are deeply embedded in the nutritional traditions and cultural identity of Central Asian peoples. Among them, kurt, a concentrated, dried sour-milk product, holds a special place as a symbol of both cultural heritage and dietary functionality [1]. Kurt is a firm, sun-dried cheese obtained from fermented and pressed milk, traditionally prepared from cow’s, goat’s, or occasionally sheep’s, camel’s, or mare’s milk. Historically, it served as a vital food source for nomadic communities due to its long shelf life, high nutrient density, and ease of storage and transport. The product is especially valuable in the dry continental climate of Kazakhstan, where its high salt content compensates for the body’s loss of electrolytes through perspiration and physical exertion [2,3].

In traditional diets, kurt is used not only as a cheese or snack but also as a seasoning and functional food. It can be consumed as a protein-rich dairy cheese, dissolved in hot water to prepare thick, nourishing soups, or used to season vegetable dishes, replacing salt-based condiments [4]. The product’s moderate acidity aids in the digestion of fatty foods, while its high protein and mineral content make it suitable for both adults and children. Kurt is also valued for its therapeutic and preventive properties—it restores intestinal microflora, stimulates digestion, and provides essential vitamins (A, E, D, C) and minerals such as calcium, magnesium, and potassium, which support bone metabolism, immunity, and cardiovascular health. In goat-milk-based kurt, the amino acid composition of proteins closely resembles that of human milk proteins, contributing to superior digestibility and bioavailability. The product’s unique composition—complete milk proteins, bioactive substances, enzymes, carbohydrates, and micronutrients—explains its nutritional and functional significance. Moreover, kurt has traditionally been used as a restorative tonic for fatigue, anemia, and periods of high physical or mental load. Its caloric value averages 260 kcal per 100 g, making it a concentrated source of energy and essential nutrients [5,6].

Growing demand for fermented dairy products, together with sustainability concerns, has increased interest in using secondary dairy streams (whey, buttermilk, skim milk) and plant processing residues (pomaces, peels, press-cakes) as functional ingredients in fermented milk formulations [7,8]. This approach supports waste reduction while enabling nutritional enhancement, particularly through improved protein quality and higher dietary fiber and antioxidant content. Reviews also note that these ingredients can influence fermentation behavior and quality attributes, so optimized inclusion levels and processing conditions are required to maintain product stability and consumer acceptability [9,10].

Research on enriching fermented dairy products with plant-based components remains relevant as a practical route to improve nutritional and biological value and to support functional food development aimed at preventing or correcting nutrient deficiencies. Recent studies demonstrate that fruit and berry processing residues can be incorporated into fermented milk foods with measurable functional benefits: apple pomace syrup, bilberry pomace, and blackcurrant pomace have been associated with higher antioxidant potential and improved nutritional profiles, while also affecting physicochemical and sensory properties, indicating the need for careful optimization of inclusion levels to maintain product acceptability [11,12,13]. In addition, research on curd and other fermented milk products reports successful enrichment using underutilized botanical resources and plant-derived fractions such as walnut leaf extract as an iodine source, feijoa pulp and peel fractions, pumpkin seeds, oilseed press-cakes, and pine nuts, generally achieving targeted enhancement with acceptable sensory quality when formulation parameters are controlled [14,15,16,17].

The study aims to develop and evaluate new kurt formulations based on secondary dairy raw materials and plant-derived ingredients, assessing their effects on the chemical composition, nutritional value, and functional–technological properties of the product.

## 2. Materials and Methods

### 2.1. Samples

**Figure 1:**
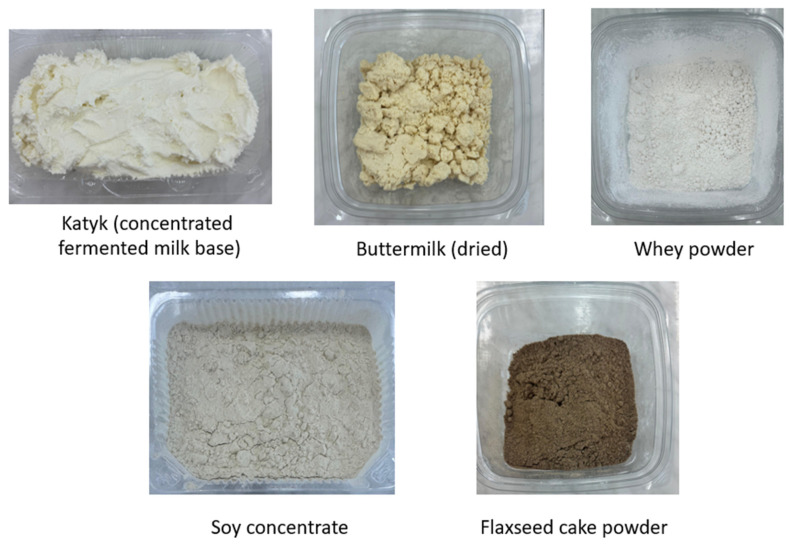
Main ingredients for the production of the fermented milk product “Kurt”.

The following raw materials were used for kurt production: skimmed milk, a mesophilic starter culture, buttermilk, curd whey, soy protein concentrate, and flaxseed cake. Skimmed milk, buttermilk, and dairy whey were supplied by the dairy processing company QazMilk (Semey, Kazakhstan). The chemical composition of the primary dairy raw materials was determined prior to formulation. Skimmed milk contained 3.4% protein, 0.2% fat, 4.9% carbohydrates, 90.8% water, and 0.8% ash. Liquid buttermilk was characterized by 3.3% protein, 10.0% fat, 13.97% carbohydrates, 72.06% water, and 0.67% ash. Flaxseed cake (30% protein, 10% fat, 10% carbohydrates) and soy protein concentrate (55% protein, 5% fat, 4% moisture) were purchased from the Aprel retail hypermarket (Semey, Kazakhstan). Figure 1 shows the main ingredients used in the production of the fermented milk product “Kurt”. Raw materials used for kurt production, including milk and starter cultures, complied with national regulatory requirements for dairy products. Supplier-provided quality certificates confirmed conformity with established microbiological and physicochemical safety standards. All materials were used within their certified shelf life.

### 2.2. Recipe Formulation and Technology for Kurt Preparation

Four experimental formulations of kurt were developed for the study, including one control and three experimental variants (Table 1). The production of kurt was carried out following a standard technological scheme with targeted modifications to incorporate additional dairy- and plant-derived protein components. Skimmed milk was filtered and pasteurized at 72–75 °C for 15–20 min, after which it was cooled to 40–42 °C. A thermophilic lactic acid starter culture (3–5%) was introduced, and fermentation proceeded at 38–42 °C for 6–8 h until a firm coagulum with a pH of 4.6–4.7 was formed. Thermophilic lactic acid fermentation was carried out using a commercial direct-vat-set (DVS) starter culture based on *Streptococcus thermophilus* and *Lactobacillus delbrueckii* subsp. *bulgaricus*, supplied by an industrial dairy culture manufacturer (Biochem s.r.l., Rome, Italy). The coagulum was separated from free whey through self-pressing or mild pressing for 10–12 h at a pressure of 10–20 kg/m^2^. The resulting concentrated fermented milk base—katyk—was stored at 4 ± 2 °C until further processing.

Auxiliary ingredients were prepared under controlled conditions. Curd whey was dried using a spray dryer with an inlet air temperature of 140–160 °C. Buttermilk protein sediment was obtained by heating buttermilk to 85–90 °C to induce protein coagulation, followed by filtration. Flaxseed cake was finely milled to ensure a homogeneous particle distribution, and soy protein concentrate was used as an industrially standardized ingredient.

The pressed katyk mass was combined with the buttermilk protein sediment, spray-dried whey, soy protein concentrate, flaxseed cake, and sodium chloride (1.0–1.5%). All components were blended until a uniform, plastic mass was formed. The mixture was shaped into spherical or tablet-like pieces using a small-scale kurt-forming device. The shaped samples were subjected to drying in a forced-air dehydrator at 35–40 °C until the final moisture content reached 10–13%.

Once dried, the product was packaged in airtight laminated pouches and stored at 0–6 °C, ensuring preservation of quality parameters for up to 12 months. This processing strategy allowed for controlled adjustment of the protein-to-fat ratio and enhancement of the nutritional profile. The integration of buttermilk protein sediment, spray-dried whey, soy protein concentrate, and flaxseed cake enabled the development of a functional dairy–plant protein matrix suitable for producing high-protein, shelf-stable fermented milk products.

### 2.3. Determination of Protein

The protein content of the dairy-based samples was determined using the Kjeldahl method, which involves quantifying total nitrogen and then converting it to crude protein using a factor of 6.25 (N × 6.25), in accordance with GOST 23327-98 [18].

### 2.4. Determination of Fat

The fat content of milk products was determined using the acid-butyrometric method in accordance with GOST 5867–2023 [19]. The procedure is based on the decomposition of the protein–fat matrix by concentrated sulfuric acid, followed by the release of fat in the presence of isoamyl alcohol. A measured portion of the sample was introduced into a butyrometer, after which sulfuric acid of the required density and isoamyl alcohol were added. The mixture was shaken until complete dissolution of proteins, thermostated at 65 ± 2 °C, and centrifuged under standardized conditions to separate the fat layer. After a second short thermostating step, the height of the fat column was read on the calibrated scale. The fat content was calculated directly from the butyrometer reading or using dilution coefficients when required. The final result was expressed as the mean of two parallel determinations meeting the repeatability criteria.

### 2.5. Determination of Ash Content

The ash content was determined according to the Pearson method. Porcelain crucibles were dried in a drying oven, cooled in a desiccator, and weighed. A 3 g portion of milk sample was placed in a crucible and incinerated in a muffle furnace at 660 °C until complete carbon removal, indicated by a gray-white residue (approximately 2 h). The crucible was then transferred to a desiccator, cooled to room temperature, and reweighed. Ash content (%) was calculated using

$$\mathrm{Ash}=\frac{B-C}{A}\times 100$$

where *A* is the sample weight (g), *B* is the weight of the crucible with ash (g), and *C* is the weight of the empty crucible (g).

### 2.6. Determination of pH

The active acidity (pH) of curd and curd-based products was determined potentiometrically in accordance with GOST R 54669-2011 [20] for milk and milk products. Approximately 5.00 ± 0.01 g of product were placed in a porcelain mortar, thoroughly mixed and triturated. Fifty milliliters of distilled water preheated to 35–40 °C were added in small portions while stirring until a homogeneous suspension was obtained. The suspension was cooled to 20 ± 2 °C and transferred to a beaker. Active acidity was measured using a pH meter equipped with combined glass and reference electrodes (measurement range 1–14 pH, accuracy ±0.02 pH). The electrodes were immersed in the suspension, which was gently stirred until a stable reading was achieved. Each sample was analyzed in duplicate under repeatability conditions, and the pH value was reported as the arithmetic mean of two measurements [20].

### 2.7. Determination of Amino Acid Composition

The amino acid composition was determined using capillary electrophoresis, a method that provides high separation efficiency without requiring prior derivatization and enables analysis of small sample volumes. Quantification of amino acids in the samples was performed using the Kapel-105M analytical system (Lumex, St. Petersburg, Russia).

A 0.5 g sample was mixed with 10 mL of hydrochloric acid solution (1:1) and subjected to hydrolytic mineralization at 110 °C for 16 h. After filtration, a 50 μL aliquot of the filtrate was evaporated to dryness under a stream of dry air in a fume hood. The dried residue was treated with a reagent for amino acid cleavage (sodium carbonate) for 35 min and again evaporated to dryness. The sample was then dissolved in 0.5 mL of distilled water and introduced into the Kapel-105M system equipped with an unmodified fused-silica capillary (inner diameter 75 μm; effective length 50 cm; total length 60 cm). Detection was carried out at 254 nm (UV). An acetate–ammonium buffer containing CuSO_4_ and β-cyclodextrin was used as the background electrolyte. Data processing was performed using Elforan software.

The mass fraction of amino acids (AA) was calculated using the following equation:

$$X=\frac{100\cdot V_{Г}\cdot V_{\mathrm{KOH}}\cdot C_{изм}}{1000\cdot m\cdot V_{aл}}$$

where *X*—mass fraction of AAs in the analyzed sample, %; $C_{изм}$—measured mass concentration of AAs in the solution, mg/dm^3^; *m*—sample mass, mg; $V_{Г}$—volume of hydrolysate used for analysis, cm^3^; *V_KOH_*—volume of the final test solution, cm^3^; $V_{aл}$—volume of hydrolysate taken for conversion of AAs to phenylthiocarbamyl derivatives, cm^3^; 100—percentage conversion factor; 1000—unit-conversion coefficient.

### 2.8. Determination of Fatty Acid Composition

Sample preparation and gas chromatographic determination of the quantitative fatty acid profile were carried out in accordance with GOST 32915-2014 [21]. Triglycerides extracted from the samples were converted to methyl esters, after which the resulting mixture was analyzed using a Khromatek–Kristall 5000 gas chromatograph (Special Design Bureau “Kromatek,” Yoshkar-Ola, Russia). Chromatographic peaks of the obtained fatty acid methyl esters were identified, and their concentrations were quantified using the internal normalization method with a 37-component standard mixture (SUPELCO).

### 2.9. Determination of Vitamin Composition (Vitamins A, D, E, and C)

The quantitative determination of vitamins in the samples was performed using standardized analytical procedures. The mass fraction of vitamin D in milk and dairy products was determined by reversed-phase high-performance liquid chromatography (HPLC) according to GOST 32916-2014 [22]. For products containing <30% fat, 20 g of sample were weighed into a 100–250 mL round-bottom flask, mixed with 25 mL of 5 mol/L KOH, 1 g of ascorbic acid and 25 mL of isopropanol, and saponified under reflux at 70–80 °C for 30 min. After cooling, the upper isopropanol layer was separated, its volume recorded, 10 mL were taken and diluted 1:1 with distilled water. Vitamin D was concentrated by solid-phase extraction on a C18 cartridge preconditioned with 50% isopropanol; the analyte was eluted with 2 mL isopropanol to obtain the test solution. For products with ≥30% fat, 10–15 g of sample were used, with the same procedure. HPLC was performed on a C18 column (4.0–4.6 × 250–300 mm, 5 µm) with acetonitrile as the mobile phase, at 23 ± 2 °C, flow rate 0.7–1.0 mL/min, injection volume 20 µL, and UV detection at 265 nm. Quantification was performed by external calibration with cholecalciferol standards (0.1–10 µg/mL); each sample was analyzed in duplicate and results expressed as the mean value.

The concentration of vitamin C (ascorbic acid) was determined in accordance with GOST 30627.2-98 [23]. The mass fraction of vitamin C (ascorbic acid) in dairy products was determined according to GOST 30627.2-98, using 2,6-dichlorophenolindophenolate (DCPIP) titration after extraction with metaphosphoric acid. Approximately 10.00 ± 0.04 g of product were weighed into a volumetric flask (50 or 100 mL, depending on the expected vitamin C level). The sample was mixed with 15–20 mL of metaphosphoric acid solution (60 g/L), shaken for at least 1 min, diluted to volume with metaphosphoric acid solution (30 g/L), thoroughly mixed and filtered. An aliquot of 10 mL filtrate was transferred to a conical flask and titrated with standardized DCPIP solution to a persistent light-pink endpoint (15 s). A reagent blank was run in parallel. The vitamin C content (mg/kg) was calculated from the titer of the DCPIP solution, the volumes of titrant used for sample and blank, the total extract volume, the titrated aliquot, and the sample mass, as specified in the standard. Each sample was analyzed in duplicate, and results were reported as the arithmetic mean.

The mass fraction of vitamin A (retinol) in dairy products was determined by the Carr–Price colorimetric method according to GOST 30627.1-98 [24]. A test portion of product (3–6 g for dry products or 15.00 ± 0.01 g for liquid products, depending on the expected vitamin A level) was placed in a round-bottom flask, mixed with distilled water (for dry products), ascorbic acid (0.10 ± 0.03 g), 40 mL ethanol and 5–7 mL of 0.5 g/mL KOH solution. The mixture was saponified under reflux in a boiling water bath for 30 ± 2 min, cooled, transferred to a separatory funnel and diluted with water. Un-saponifiable matter was extracted three times with 50 mL portions of petroleum or diethyl ether, the combined extracts were washed to neutrality, dried over anhydrous sodium sulfate and the solvent was evaporated under reduced pressure. The residue was dissolved in 2–3 mL chloroform. An aliquot (0.4 mL) was reacted with 4.0 mL antimony trichloride reagent, and the optical density was measured at 620 nm against chloroform. Vitamin A content was calculated from a calibration curve constructed with retinol standard solutions.

The mass fraction of vitamin E (α-tocopherol) in dairy products was determined by a colorimetric method according to GOST 30627.3-98 [25]. Liquid and semi-solid samples (15.00 ± 0.01 g) or an appropriate mass of dry product (2–10 g, depending on the expected vitamin E level) were mixed with ethanol, ascorbic acid (antioxidant) and 0.5 g/mL KOH solution. The mixture was saponified under reflux in a boiling water bath for 30 min, cooled, diluted with water and transferred to a separatory funnel. Un-saponifiable matter containing tocopherols was extracted three times with diethyl or petroleum ether. The combined organic phases were washed to neutrality with water, dried over anhydrous sodium sulfate and the solvent was evaporated under reduced pressure. The residue was immediately dissolved in absolute ethanol and an aliquot (1–5 mL) was reacted with ethanolic FeCl_3_ and ortho-phenanthroline (or bathophenanthroline/α,α-dipyridyl) in the presence of phosphoric acid. After color development, absorbance was measured at 490 nm against an ethanol blank. Vitamin E concentration was obtained from a calibration curve prepared with α-tocopherol standards and expressed per mass of product. Each sample was analyzed in duplicate.

### 2.10. Mineral Composition Analysis

The concentrations of calcium, sodium, potassium, and magnesium were determined using atomic absorption spectrometry in accordance with ISO 8070:2007 [26].

### 2.11. Water-Holding Capacity (WHC)

The water-holding capacity of the fermented milk samples was determined using a standardized centrifugation method. Approximately 20 g of each sample (m_1_) were cooled to 4 °C and stored for 24 h, after which they were centrifuged at 3000 rpm for 10 min at 20 °C. The released whey (m_2_) was removed and weighed [27].

The WHC (%) was calculated using the following equation:

$$WHC=\frac{m_{1}-m_{2}}{m_{1}}\times 100$$

where *m*_1_—initial mass of the fermented milk sample (g); *m*_2_—mass of the separated whey (g).

### 2.12. Determination of Water Activity

Water activity (a_w_) was measured using an aWLife analyzer (Steroglass S.r.l., Perugia, Italy). Prior to analysis, the instrument was preheated and prepared for operation according to the manufacturer’s recommendations. The prepared sample was placed into the measuring chamber, and analysis was initiated by pressing the “START” button on the main screen. Before each new measurement, the sample name was entered, and the “START” command activated the thermostating process. The display status changed from READY to SAMPLE THERMOSTATTING. Once the set temperature was reached, the status changed to ANALYSIS. After completion, the device emitted several sound signals and displayed the final results. Calibration was performed based on the obtained measurement values. The “CALIBRATION” menu was used, followed by “SELECTIVE CALIB,” where appropriate reference standards were selected to verify measurement accuracy. Upon completion of calibration, the “OPTIMIZE” function was activated to enhance instrument performance. Water activity of the test samples was subsequently measured. The device recorded the a_p_w value after equilibrium was reached. All measurements were conducted at 25 °C.

### 2.13. Measurement of Color Characteristics of Food Products

Color parameters were assessed using a Chroma Meter CR-400 (Konica Minolta Inc., Tokyo, Japan), designed for determining color characteristics in the CIE Lab* system. Before measurement, the device was allowed to stabilize for several minutes. Standard calibration tiles (white and, if necessary, dark) were prepared. The calibration tile was placed over the measurement aperture, and calibration was performed using the “Calibration” mode on the device menu. When required, additional calibration on black or green reference tiles was conducted.

Sample surfaces were prepared to be smooth, clean, and free of glare. The instrument was positioned so that the measurement aperture was in full contact with the sample surface, after which measurements were taken. Results were recorded in CIE Lab* coordinates:

L*—lightness (0 = black, 100 = white);
a*—green (negative) to red (positive) axis;
b*—blue (negative) to yellow (positive) axis.

Additional color parameters, including total color difference (ΔE), chroma (C*), and hue angle (h°), were calculated when necessary.

This method provides an accurate and objective evaluation of the color characteristics of the food ingredient.

### 2.14. Sensory Analysis

Sensory evaluation of the kurt product was carried out by a trained panel consisting of 11 members from the Laboratory of Food Analysis of Shakarim University with prior experience in the sensory assessment of dairy products. The analysis was conducted in a sensory evaluation room under controlled conditions of temperature (22 ± 2 °C) and neutral lighting. Samples were coded with random three-digit numbers and presented to panelists in a randomized order to minimize bias. The sensory attributes evaluated included appearance, color, texture, aroma, taste, and overall acceptability. Each attribute was assessed using a structured five-point descriptive–hedonic scale, where verbal descriptors were assigned numerical values as follows: 1—very poor, 2—poor, 3—acceptable, 4—good, and 5—very good. Panelists rinsed their mouths with water between samples. For data processing, the verbal sensory responses were converted into numerical scores according to the defined scale. Mean values and standard deviations were calculated and used for statistical analysis and comparison of samples [28].

### 2.15. Microbiological Analysis

Microbiological quality of the kurt samples was evaluated using standard culture-based methods. Before analysis, samples were aseptically homogenized with sterile peptone water (1:9, *w*/*v*) and serial decimal dilutions were prepared. Total viable count (TVC) was determined by pour-plating on Plate Count Agar and incubating at 30 °C for 72 h. Lactic acid bacteria were enumerated on de Man–Rogosa–Sharpe (MRS) agar after incubation at 37 °C for 48 h under anaerobic conditions. Yeasts and molds were determined on Sabouraud dextrose agar supplemented with chloramphenicol, incubated at 25 °C for 5 days. The presence of pathogenic microorganisms, including *Escherichia coli*, *Salmonella* spp., and *Staphylococcus aureus*, was assessed according to relevant ISO standards for dairy products. Microbiological results were expressed as colony-forming units per gram of sample (CFU g^−1^) [29].

### 2.16. Statistics

The production of kurt was carried out in independent technological replicates. Each formulation was prepared in triplicate in separate production batches, including milk fermentation, curd processing, shaping, and drying. All sensory and physicochemical analyses were performed in triplicate (*n* = 3), and results are expressed as mean ± standard deviation. Differences among the control and experimental formulations were evaluated using one-way analysis of variance (ANOVA). When significant effects were detected, Tukey’s HSD post hoc test was applied for pairwise comparisons. A significance level of *p* < 0.05 was used to determine statistically meaningful differences. All statistical processing and graphical outputs were generated using Microsoft Excel 2016 (Microsoft Corp., Redmond, WA, USA) and Statistica 12 PL (StatSoft, Tulsa, OK, USA).

## 3. Results

### 3.1. Chemical and Amino Acid Composition of Buttermilk and Spray-Dried Whey

The study of the chemical and amino acid composition of buttermilk protein sediment and spray-dried whey provides insight into the nutritional potential of these secondary dairy resources as functional ingredients for kurt production. Both components exhibit complementary nutritional profiles that together form a balanced base for developing composite fermented dairy products with improved protein and mineral value.

The buttermilk protein sediment demonstrated a rich and balanced macronutrient composition, characteristic of coagulated protein–lipid systems. The moisture content was 61.45%, indicating a high proportion of bound water typical for thermally coagulated milk proteins (Table 2). The protein content reached 11.51%, while the fat content was 17.78%, reflecting the concentration of residual milk fat globules associated with phospholipid and protein complexes. The carbohydrate and ash contents were 7.53% and 1.73%, respectively. This profile confirms that buttermilk sediment represents a protein–lipid concentrate containing both bioavailable proteins and essential mineral fractions, suitable for fortification of fermented dairy products.

The amino acid profile of buttermilk protein sediment revealed the presence of all essential amino acids necessary for human nutrition. The dominant amino acids included proline (1.83%), leucine and isoleucine (1.34%), lysine (1.22%), and valine (1.22%), which together ensure a high biological value of the protein fraction (Table 3). Amino acids such as threonine (1.02%), serine (1.06%), and arginine (1.18%) were also present in considerable amounts, contributing to the balanced amino acid ratio. The presence of methionine (0.61%) and phenylalanine (0.81%) further emphasizes the nutritional completeness of this matrix. The high content of proline and glycine suggests structural stability of the coagulated mass and potential improvement of texture in composite food systems.

In contrast, the spray-dried whey exhibited a markedly different macronutrient profile dominated by carbohydrates. The carbohydrate fraction accounted for 73.92%, primarily due to lactose, which imparts a naturally sweet taste and strong water-binding properties. The protein content was 10.08%, fat—0.67%, ash—4.79%, and moisture—9.47%. These values are typical of high-quality dried whey powders and reflect their role as energy-rich, mineral-rich ingredients with moderate protein content. The relatively high ash content indicates the preservation of essential macroelements such as calcium and magnesium, contributing to the functional and mineral value of whey as a fortifying additive.

The amino acid composition of spray-dried whey also demonstrated a complete set of essential amino acids, though at lower absolute levels compared to buttermilk sediment. The major amino acids were proline (1.31%), leucine and isoleucine (0.91%), valine (0.77%), threonine (0.83%), and lysine (0.76%). The concentrations of arginine (0.61%), methionine (0.32%), and phenylalanine (0.47%) were lower, consistent with the reduced protein fraction after drying. Despite this, the amino acid balance remained favorable for nutritional applications. The relatively high proline content supports desirable structural properties, while serine (0.65%) and alanine (0.52%) contribute to mild flavor and digestibility.

Comparative analysis indicates that buttermilk sediment provides a protein- and lipid-rich substrate with high biological value, while spray-dried whey offers a carbohydrate- and mineral-rich matrix with good solubility and sweet taste. The higher total amino acid content of buttermilk, particularly in lysine, leucine, and proline, enhances its role as a structural and nutritional enhancer. Conversely, whey contributes lactose and minerals, improving flavor, energy density, and rehydration properties.

### 3.2. Studying the Food Value of Concentrated Fermented Milk Product—Katyk

#### 3.2.1. Studying the Chemical Composition of Katyk

Katyk produced from skimmed milk through traditional fermentation exhibited a well-balanced composition of essential nutritional components. The moisture content was 69.48%, protein 18.83%, fat 3.91%, carbohydrates 5.06%, and ash 1.42% (Table 4). The elevated protein level compared to raw milk is attributed to concentration effects caused by partial moisture loss and the proteolytic activity of starter cultures, which promote the accumulation of peptides and free amino acids. The low fat content reflects the use of skimmed raw material, contributing to a light texture and dietetic character of the product.

#### 3.2.2. Studying the Amino Acid Composition of Katyk

Amino acid analysis demonstrated that katyk possesses a complete and balanced amino acid profile. The highest concentrations were observed for proline (3.03%), leucine and isoleucine (1.95%), lysine (1.87%), and valine (1.66%). Significant levels of tyrosine (1.51%), serine (1.44%), and threonine (1.15%) provide an optimal ratio of essential to nonessential amino acids. The high concentrations of proline and glycine contribute to the formation of the typical dense texture of katyk and improve its rheological properties (Table 5).

#### 3.2.3. Vitamin and Mineral Composition of Katyk

The vitamin profile of katyk included vitamin A (0.046 mg/100 g), vitamin E (0.22 mg/100 g), and vitamin C (0.38 mg/100 g). Despite partial degradation of thermolabile compounds during pasteurization, the product retained physiologically relevant concentrations of antioxidant vitamins. Vitamin E plays a protective role by preventing oxidative degradation of lipids, while vitamin C enhances the overall antioxidant potential and exhibits a synergistic effect with vitamin E.

The mineral composition of katyk was characterized by a high content of calcium (309.4 mg/100 g) and magnesium (27.81 mg/100 g), confirming its value as a source of essential macroelements (Table 6). The high calcium level results from the preservation of protein–mineral complexes during fermentation, whereas the presence of magnesium facilitates calcium absorption and supports metabolic balance.

Overall, katyk produced from skimmed milk is distinguished by its high protein content, balanced amino acid composition, bioavailable calcium and magnesium forms, and moderate levels of antioxidant vitamins. These attributes confirm its high nutritional and biological value and underscore its potential for use in functional fermented dairy products, including kurt enriched with plant-based and protein–mineral additives.

### 3.3. The Chemical Composition Analysis of the Kurt

The chemical composition of the kurt samples showed clear formulation-dependent differences in moisture, ash, protein, fat, and carbohydrate contents, demonstrating the impact of dairy- and plant-derived ingredients on the nutritional profile. Moisture ranged from 11.68 to 13.61%, with the control showing the highest value. All experimental variants had significantly lower moisture levels (*p* < 0.05), indicating more efficient dehydration and denser matrix formation due to reduced water-binding capacity from whey and plant components. Ash content varied between 7.95 and 9.56%. The control contained the highest ash level, consistent with the natural mineral density of fermented milk solids. The experimental samples showed slightly reduced ash values (*p* < 0.05), reflecting dilution caused by carbohydrate-rich additives such as spray-dried whey and flaxseed cake, which contain proportionally less mineral matter.

Protein exhibited the most pronounced differences. The control contained 52.04% protein, whereas the experimental variants showed lower levels: 34.41% (Experiment 1), 40.99% (Experiment 2), and 30.67% (Experiment 3). These differences (*p* < 0.05) resulted from replacing part of the high-protein katyk base with whey powder and plant materials (Table 7). Experiment 2 achieved the highest protein level among enriched variants due to its greater proportion of buttermilk protein sediment, which strengthened the protein matrix and produced a more favorable macronutrient balance.

Fat content ranged from 7.8 to 12.05%. Experiment 2 displayed the highest fat level (*p* < 0.05), likely due to lipid fractions contributed by buttermilk protein sediment and soy concentrate. Experiment 3 demonstrated the lowest fat content, linked to reduced buttermilk addition and the presence of flaxseed cake, whose fiber components may bind lipids and limit extraction.

Carbohydrates varied inversely with protein, from 13.99% in the control to 41.85% in Experiment 3. Higher carbohydrate values in Experiments 1 and 3 (*p* < 0.05) reflect increased lactose from whey powder and polysaccharides from plant ingredients, enhancing energy density but reducing relative protein concentration.

The elevated protein-to-fat ratio observed in the developed kurt formulations (up to approximately 5:1) reflects the specific production technology and intended use of kurt as a concentrated fermented milk product, rather than as a conventional staple food. Kurt is traditionally consumed as a functional dairy snack or supplement, where protein density is prioritized over lipid content. The predominance of the protein fraction (up to ~52%) relative to fat (≤12%) is technologically justified, as excessive fat impedes effective moisture removal during drying and can compromise shelf life due to oxidative fat deterioration. Consequently, maintaining a moderate fat level is essential for achieving low water activity, structural stability, and long-term storability. This compositional profile corresponds to traditional physicochemical characteristics of kurt and is consistent with existing regulatory standards, which define kurt as a high-protein fermented dairy product.

Overall, formulation adjustments produced targeted shifts in nutritional composition. Experiment 2 showed the most balanced profile, combining elevated protein (40.99%) and fat (12.05%) with moderate ash and carbohydrate levels. These results confirm that dairy and plant ingredients can modify the macronutrient profile of kurt when used within optimized ratios, while maintaining its protein-dense character.

### 3.4. The Physicochemical Parameters (pH and Water Activity)

**Figure 2:**
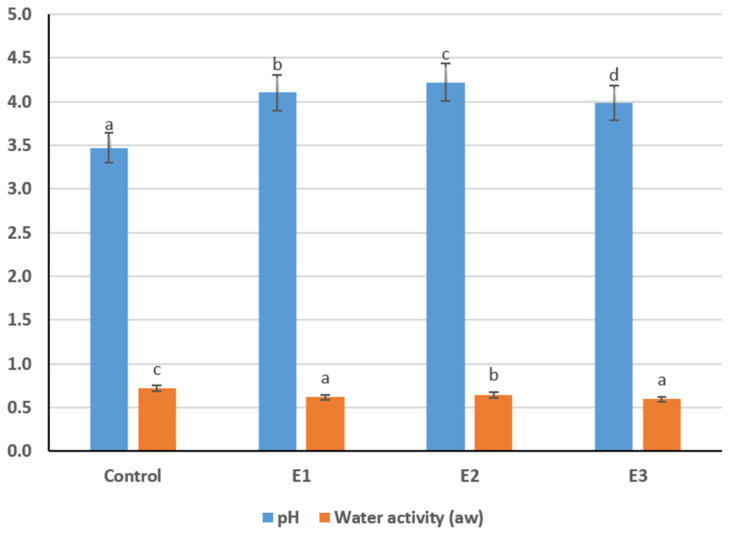
pH and water activity of kurt samples (Different lowercase letters (a–d) indicate statistically significant differences within the same indicator (*p* < 0.05)).

The physicochemical assessment showed that formulation changes affected both pH and water activity (aw), two key indicators of microbial stability and textural quality in dried fermented milk products (Figure 2). pH values ranged from 3.47 in the control to 4.22 in Experiment 2. The control sample showed the greatest acidity, reflecting typical lactic fermentation. All experimental variants exhibited higher pH values (*p* < 0.05), indicating a buffering effect from added protein- and plant-based components. Ingredients such as buttermilk protein sediment and soy concentrate contain amino and phosphate groups that bind hydrogen ions, moderating acidity [30]. Experiment 2 reached the highest pH (4.22; *p* < 0.05), consistent with its higher proportion of buttermilk protein sediment and its balanced macronutrient profile. E1 (4.10) and E3 (3.98) also demonstrated elevated pH relative to the control, though to a lesser extent. These moderate increases are technologically beneficial, as they may prevent excessive sourness and reduce brittleness associated with strong acid gels [31].

Water activity ranged from 0.5954 to 0.7193, with the control showing the highest aw (0.7193). All enriched samples exhibited significantly reduced aw (*p* < 0.05), indicating stronger water immobilization and enhanced dehydration. Experiment 3 had the lowest aw (0.5954), reflecting its higher flaxseed cake content, as flaxseed fibers bind water and reduce free moisture. E1 (0.6169) and E2 (0.6453) also showed substantially lower aw compared to the control, confirming improved matrix consolidation.

Overall, the combined decrease in aw and moderate rise in pH indicate that enriched formulations maintain favorable technological properties and microbial stability. Low water activity ensures extended shelf life, while controlled acidity supports acceptable sensory characteristics in functional dairy–plant kurt products.

### 3.5. Water-Holding Capacity of Kurt Samples

The evaluation of water-holding capacity (WHC) provides important insight into the structural stability and quality of kurt formulations. These parameters determine the product’s texture, yield, and overall sensory characteristics, and they are directly influenced by the composition and interaction of protein, fat, and carbohydrate components within the matrix [32,33,34].

**Figure 3:**
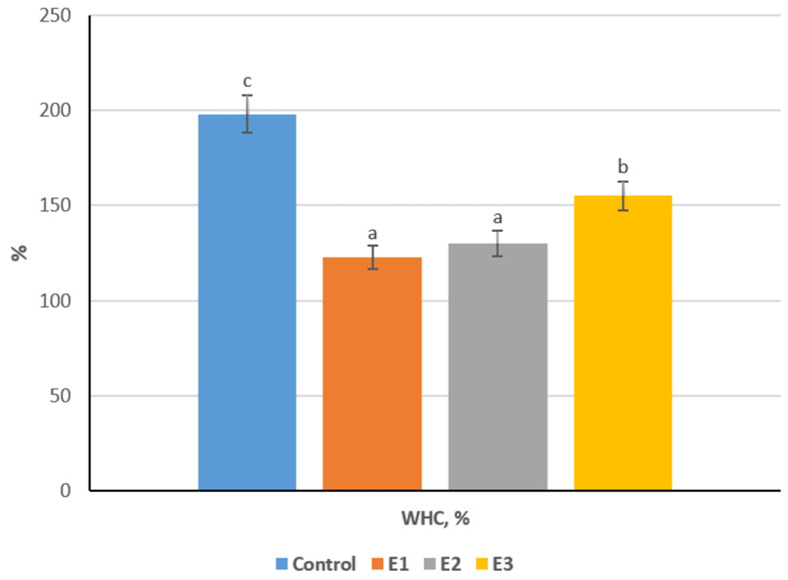
WHC of kurt variants; different lowercase letters (a–c) indicate statistically significant differences within the same indicator (*p* < 0.05).

The water-holding capacity (WHC) of the samples varied considerably, ranging from 123% to 198% (Figure 3). The control sample exhibited the highest WHC (198%), which can be attributed to the predominance of coagulated milk proteins and the absence of additional dry components that could reduce hydration capacity. In contrast, the introduction of spray-dried whey, soy concentrate, and flaxseed cake in the experimental variants led to a marked decline in WHC (*p* < 0.05) compared to the control, indicating that the partial replacement of katyk with dry or fibrous ingredients reduced the overall water-binding potential of the protein network.

Among the experimental formulations, Experiment 3 showed the highest WHC (155%; *p* < 0.05), which can be explained by the presence of 10% flaxseed cake, known for its content of hydrophilic polysaccharides and mucilage capable of retaining bound water. The moderate improvement of WHC in Experiment 3 relative to Experiment 1 (123%) and Experiment 2 (130%) suggests that the synergistic interaction between milk proteins and plant polysaccharides enhances matrix hydration, even though total water-binding remained below the control level. Experiment 1 demonstrated the lowest WHC (123%; *p* < 0.05), likely due to the relatively high proportion of dry whey (18%) and lower amount of buttermilk protein sediment, resulting in a denser, less porous structure with reduced capacity to immobilize water.

The reduction in a_w_ and WHC in enriched kurt formulations is not solely a dilution effect but is largely governed by molecular interactions between milk proteins and plant-derived polysaccharides and fibers. The incorporation of soy proteins, whey components, and flaxseed cake promotes protein–polysaccharide interactions, including hydrogen bonding, electrostatic interactions, and physical entrapment of water within a more consolidated biopolymer network. These interactions increase the proportion of bound water while reducing free, mobile water, thereby lowering a_w_ and modifying WHC.

These findings support the research hypothesis that the integration of buttermilk protein sediment and plant-derived ingredients can modulate the functional–technological characteristics of kurt, enabling the development of formulations with tailored hydration and emulsification behavior suited for high-protein, shelf-stable functional dairy–plant products.

### 3.6. Amino Acid Composition

Across the formulations, most amino acids remained within a comparable range; however, several components showed clear deviations from the control. The combined proportion of leucine and isoleucine decreased from 10.84% in the control to 7.45% in Experiment-2 and 6.86% in Experiment-3, while the summed values for Experiment-1 (leucine 2.67% + isoleucine 1.62%) reached only 4.29%. Thus, all composite samples demonstrated a noticeable decline in these amino acids, with the smallest reductions observed in Experiment-2 and Experiment-3. This decrease reflects the reduced proportion of casein-rich katyk and its replacement by whey and plant proteins, which inherently contain lower levels of these residues (Table 8). Proline exhibited a similar pattern. Its concentration declined from 16.87% in the control to 13.18% in Experiment-1, 12.94% in Experiment-2, and 12.42% in Experiment-3. Although proline remained the most abundant amino acid in all samples, the consistent reduction highlights the diminished contribution of casein-derived structures in the composite formulations. Differences among the experimental variants were relatively small, indicating that once casein is diluted, further adjustments in ingredient ratios exert only incremental effects on proline proportion.

In contrast, several amino acids showed minimal changes relative to the control. Lysine remained stable (10.44% in control vs. 8.87–8.99% in experiments), and valine shifted only slightly (9.24% in control vs. 7.65–8.13% in experiments). Threonine (6.43% vs. 5.90–6.22%), methionine (4.82% vs. 3.37–3.69%), and phenylalanine (5.82% vs. 5.61–5.68%) also remained within a narrow interval across all variants. Tyrosine decreased from 8.43% in the control to 6.11–6.84% in the experiments, whereas serine (8.03% vs. 7.04–7.42%), arginine (7.03% vs. 7.21–7.39%), and histidine (4.62% vs. 4.03–4.16%) changed only slightly. Aspartic acid, glutamic acid, and cysteine—absent in the control report—were present in all composite samples (Asp 3.11–4.24%, Glu 5.47–7.03%, Cys 0.43–0.52%) and varied minimally among formulations.

Overall, the most notable changes relative to the control were reductions in leucine–isoleucine and proline, both of which were associated with the lower proportion of casein-containing katyk. Among the experimental variants, Experiment-2 exhibited the most favorable balance: it maintained higher combined leucine–isoleucine levels than Experiment-1 (7.45% vs. 4.29%) while preserving overall amino acid stability across the remainder of the profile.

### 3.7. Vitamin Composition of Kurt Samples

**Figure 4:**
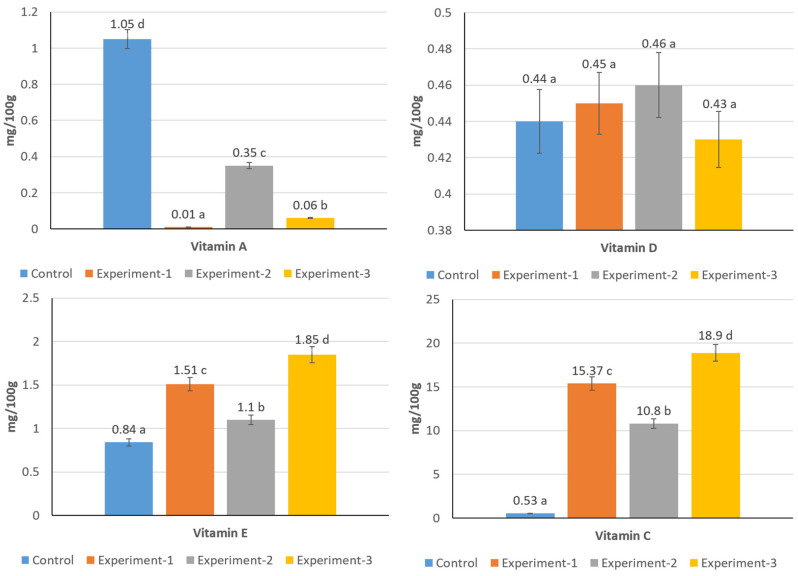
Vitamin concentration in kurt samples (Different lowercase letters (a–d) indicate statistically significant differences between different samples (*p* < 0.05)).

The vitamins that appeared to be present at nutritionally relevant levels in the formulations, based on their contribution from the individual ingredients, were quantified in the control and experimental kurt samples. The analyses showed clear differences among the variants, particularly for vitamins A, E, and C, while vitamin D remained relatively stable across samples (Figure 4). The control sample contained a notable amount of vitamin A (1.05 mg/100 g), whereas all experimental formulations showed markedly lower values. The lowest content was observed in E1 (0.01 mg/100 g), with similarly low levels in E3 (0.06 mg/100 g). E2 showed a slightly higher concentration (0.35 mg/100 g), although still well below the control. These results indicate that only the control sample provided vitamin A in significant amounts, while its contribution in the composite formulations was minimal.

Vitamin D content was comparable across all samples, ranging from 0.43 to 0.46 mg/100 g. The similarity of these values suggests that the inclusion of buttermilk protein sediment, whey, soy, and flax ingredients did not substantially influence the retention of this vitamin, and all kurt variants maintained similar levels.

In contrast, vitamin E showed higher levels in the experimental samples than in the control (0.84 mg/100 g). E3 presented the greatest amount (1.85 mg/100 g), followed by E1 (1.51 mg/100 g) and E2 (1.10 mg/100 g). Thus, vitamin E reached significant levels only in the composite formulations, reflecting the contribution of flaxseed cake and soy concentrate.

The most pronounced differences were found in vitamin C. While the control sample contained only 0.53 mg/100 g, all experimental variants showed substantially higher concentrations. E1 reached 15.37 mg/100 g, E2 contained 10.8 mg/100 g, and E3 showed the highest value at 18.9 mg/100 g. These results demonstrate that vitamin C was present in significant amounts exclusively in the composite formulations, with E3 providing the highest level.

Overall, the vitamin composition of kurt was strongly influenced by the formulation. The control showed significant levels of vitamin A, whereas the composite samples provided considerable amounts of vitamins E and C. Vitamin D remained largely unchanged across all variants. Among the experimental formulations, E3 exhibited the most favorable profile, with the highest levels of vitamins E and C, indicating enhanced antioxidant potential in this variant.

### 3.8. Mineral Composition of Kurt Samples

The analyses showed that minerals (calcium and magnesium) were present in appreciable amounts, although their concentrations varied depending on the composition of each formulation (Table 9). Calcium content ranged from 732.8 to 880.7 mg/100 g, with the control sample containing 750 mg/100 g. Both E1 (856 mg/100 g) and E3 (880.7 mg/100 g) showed higher calcium levels than the control, indicating that these formulations provided a significant amount of this mineral. In contrast, calcium in E2 (732.8 mg/100 g) was slightly lower than in the control. These differences are consistent with the varying proportions of buttermilk protein sediment and spray-dried whey, which are known to contribute calcium-rich components. Thus, E1 and E3 showed appreciable enrichment, while E2 showed a modest reduction relative to the control.

Magnesium levels followed a similar pattern. While the control contained 38.5 mg/100 g, all experimental samples presented higher values: 85.5 mg/100 g in E1, 71.1 mg/100 g in E2, and 88.4 mg/100 g in E3. These findings suggest that magnesium was present in significant amounts only in the composite formulations, likely reflecting the contribution of flaxseed cake and soy concentrate, both of which are rich in magnesium-containing compounds. The highest values were observed in E3, followed closely by E1.

Overall, the mineral composition of kurt varied according to the formulation. E3 showed the highest levels of both calcium and magnesium, whereas E1 also demonstrated substantial enrichment. E2 showed moderate concentrations of both minerals. These results indicate that the integration of dairy and plant components can influence mineral levels in kurt, with certain combinations—particularly those in E3—providing enhanced mineral content that may be beneficial in functional food applications.

### 3.9. Sensory Evaluation

**Figure 5:**
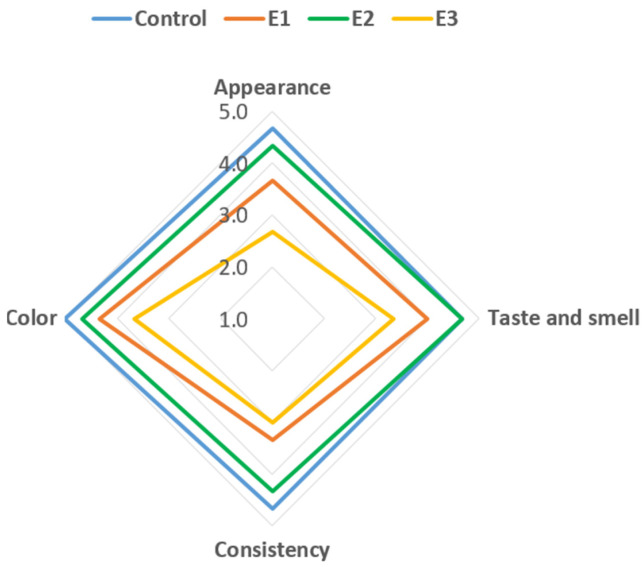
Sensory profile of different variants of kurt samples.

Sensory evaluation demonstrated that modification of kurt formulations with whey- and flaxseed-based ingredients produced distinct and statistically significant effects on key sensory attributes (*p* < 0.05) (Table 10). The findings clarify how far the formulation can be altered while preserving the traditional sensory profile, thereby directly addressing the research objective of developing nutritionally enriched yet acceptable kurt variants (Figure 5).

**Figure 6:**
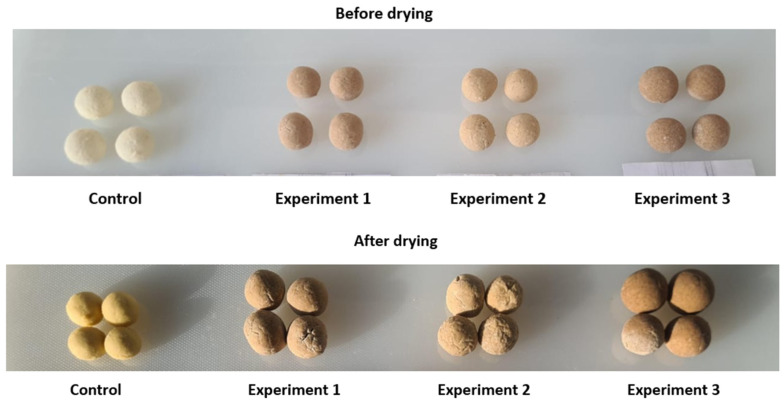
Appearance of kurt samples before and after drying.

Appearance. All samples retained the characteristic spherical form, indicating that added ingredients did not disrupt shaping or drying (Figure 6). However, appearance scores differed markedly. The control showed the highest acceptability (4.67 points), while E1 (3.67) and especially E3 (2.67) exhibited significant declines (*p* < 0.05). These decreases corresponded to color changes from the control’s light-yellow to progressively darker shades. Moderate browning in E1 and E2 was tolerated (E2: 4.33 points), but the dark-brown surface of E3 strongly reduced acceptability (*p* < 0.05).

Taste and smell. Taste and odor were the strongest discriminating factors. The control and E2 shared the traditional “sour milk, slightly salty” profile and both received the highest scores (4.67), indicating successful preservation of characteristic flavor. E1 showed a mild decrease (4.0; *p* < 0.05), while E3 exhibited the lowest taste score (3.33; *p* < 0.05), associated with pronounced flaxseed- and whey-derived notes. These results show a dose-dependent effect: low-intensity non-traditional flavors are acceptable, but stronger notes reduce hedonic perception.

Consistency. All samples were hard and dry as expected for kurt, but structural coherence varied. The control scored 4.67, and E2 remained close (4.33; *p* < 0.05). E1 (3.33) and E3 (3.0) showed significantly poorer texture (*p* < 0.05), with E3 described as crumbly. This indicates that higher inclusion levels of added ingredients can disrupt matrix uniformity.

Color. Color scores aligned with appearance results. The control received 5.0 points, while E2 (4.67) and E1 (4.33) showed acceptable decreases (*p* < 0.05). E3’s dark-brown hue resulted in a much lower score (3.67; *p* < 0.05), reinforcing that excessive darkening is sensory-negative.

Across all attributes, E2 demonstrated the best balance between added functionality and sensory acceptability. Although some differences from the control were statistically significant, they were minor in practical terms. In contrast, E1 and especially E3 showed consistent reductions in appearance, taste, aroma, texture, and color, indicating that excessive modification leads to pronounced deviations from the typical sensory profile of kurt.

These results confirm that nutritionally enriched kurt can be developed without compromising sensory quality, provided that ingredient levels remain within an optimal range exemplified by the E2 formulation.

### 3.10. Microbiological Quality and Safety Assessment of the Experimental Kurt Formulations

In the control sample, the total mesophilic aerobic microbial count (TMAMC) was 1.0 × 10^3^ CFU/g, which is typical for traditional kurt characterized by low moisture content and pronounced acidity. The incorporation of dried curd whey in Experiment 1 led to a slight increase in TMAMC to 1.2 × 10^3^ CFU/g, likely due to the additional fermentable carbohydrates and minerals that may support residual microflora. In Experiment 2, which contained a combination of dried whey and buttermilk protein sediment, the microbial count decreased to 9.0 × 10^2^ CFU/g. This reduction may be attributed to the stronger acidification and the presence of bioactive peptides with antimicrobial properties. The lowest value was recorded in Experiment 3 (6.0 × 10^2^ CFU/g), enriched with soy protein concentrate and flaxseed cake (Table 11). These plant-derived components, known for their moisture-binding capacity and phenolic compounds, likely limited microbial proliferation. All TMAMC values remained well below the regulatory limit of 5.0 × 10^3^ CFU/g, confirming the microbiological safety of the samples.

Yeast counts in all samples were markedly below the permissible level (≤50 CFU/g). The control sample contained 10 CFU/g. Experiment 1 showed a minor increase to 15 CFU/g, which may be associated with the availability of additional fermentable substrates introduced by dried curd whey. Experiments 2 and 3 exhibited reduced yeast levels (8 and 5 CFU/g, respectively), reflecting the effects of increased acidity, bioactive compounds present in buttermilk, and the antifungal potential of flaxseed constituents combined with reduced water activity in formulations containing plant-based ingredients.

Mold counts also complied with the regulatory requirement (≤100 CFU/g). The control contained 10 CFU/g. Experiment 1 demonstrated a moderate increase to 20 CFU/g, potentially due to incidental introduction of spores from powdered whey. Mold levels in Experiment 2 (12 CFU/g) remained comparable to the control, whereas the addition of soy protein concentrate and flaxseed cake in Experiment 3 resulted in a decline to 7 CFU/g, likely due to the inhibitory effects of phenolic compounds and reduced water availability.

Pathogenic microorganisms such as coliforms, *Staphylococcus aureus*, *Listeria monocytogenes*, and *Salmonella* were not detected in any of the samples.

## 4. Discussion

The development of fortified and functionally enhanced kurt requires a clear understanding of how formulation changes influence its chemical, nutritional, and microbiological properties. The present findings demonstrate several pathways for solving the central problem: improving the nutritional density of kurt while preserving its safety and sensory integrity. By examining chemical composition, physicochemical behavior, bioactive components, mineral and vitamin enrichment, and microbial stability, this work identifies the mechanisms through which dairy- and plant-derived ingredients reshape the product’s structural and functional matrix. The results provide scientific insight into how milk by-products and plant materials interact with fermented milk solids, influencing water binding, buffering capacity, nutrient distribution, and microbial resilience. Such integration of compositional and functional data offers a framework for rational formulation design, enabling controlled modification of traditional products while maintaining technological feasibility. These insights contribute to the broader scientific effort to create stable, nutrient-dense, culturally rooted fermented foods using sustainable raw materials and evidence-based processing strategies.

In terms of chemical composition, substantial differences are observed between the current findings and those reported for traditional Turkish kurut (kurt). Patır and Ateş described products with high fat (32.9%) and ash (11.79%) contents as well as moderate moisture (10.96%) and carbohydrate levels [36], whereas the present formulations demonstrated considerably lower fat (7.80–12.05%) and ash (7.95–9.56%) but markedly higher protein values (30.67–52.04%). Similar differences were noted when compared with samples analyzed by Atasever and Atasever [37], whose kurut displayed fat levels of 16.7–22.6% and slightly variable protein. Studies focusing on drying technologies, such as those by Anlı [38] and by Yıldız & Bayat [39], reported physiochemical ranges typical for traditional products: fat 8.2–8.9%, protein 44.4–53.6%, and dry matter 80.3–95%, depending on the method. Within this compositional range, differences between the control and modified variants reflect formulation-driven macronutrient redistribution rather than uniform protein enhancement. All experimental variants exhibited lower protein levels than the control (30.67–40.99% vs. 52.04%), reflecting partial replacement of the casein-rich katyk base with whey powder and plant ingredients. This substitution introduced additional lactose and plant polysaccharides, leading to a reciprocal increase in carbohydrate content and a dilution of relative protein concentration, particularly in Experiments 1 and 3. Nevertheless, the formulations differed in protein quality and balance: Experiment 2 retained the highest protein level among modified variants due to its higher proportion of buttermilk protein sediment, resulting in a more balanced macronutrient profile rather than absolute protein enrichment.

Regarding pH and water activity, earlier works typically reported pH values around 4.01–4.35 for naturally fermented kurut, consistent with acid-coagulated dairy matrices. These ranges align with those found in the present analysis (3.47–4.22). However, water activity is rarely reported in published studies. Traditional products dried under sun or oven conditions, as described by [36] and [39], generally exhibit moisture-based dryness but lack specific aw measurements. The very low water activity values (0.595–0.719) reported here provide a more precise reflection of product stability and help contextualize the enhanced microbial safety of the formulations compared to the literature, where microbial presence is often substantial despite low moisture.

The observed amino acid shifts have practical nutritional implications because they indicate a change in protein composition rather than simply total protein level. Reduced leucine–isoleucine and proline suggest dilution of casein-derived proteins as katyk is partially replaced by whey and plant ingredients, which can decrease the relative contribution of branched-chain amino acids associated with muscle protein maintenance. At the same time, the near-stability of lysine, valine, threonine, methionine, and phenylalanine indicates that key essential amino acids remain broadly preserved, with E2 maintaining the most balanced pattern among the modified variants. Amino acid composition has rarely been addressed in kurut literature. Studies such as those by İstanbullugil et al. [40] evaluated phenolic and flavonoid characteristics of plant extracts added to kurut rather than amino acid profiles of the product itself. Consequently, the detailed amino acid profiling in the present research extends beyond previous works and highlights improved nutritional density associated with enriched formulations based on whey, buttermilk sediment, soy, and flaxseed ingredients.

Mineral composition varied widely across studies. The X-ray microanalysis performed by Omaralieva et al. [41] demonstrated that traditional market kurut contained high concentrations of sodium (20.8–26.4%) and chlorine (51.6–54.8%), reflecting heavy salting practices, while calcium ranged from 6.08% to 8.72% and potassium from 3.07% to 6.29%. These findings differ from the balanced mineral distribution observed in the current formulations, where fortification strategies and controlled ingredient selection increased calcium retention and moderated overall salinity. The kelp-enriched “biokurt” developed by Sherova et al. [42] presented an alternative mineral enhancement strategy, where iodine content increased from 85.9 to 119.5 µg/kg with 0.5% Laminaria addition. Although effective for iodine fortification, that study did not assess broader nutrient interactions or microbiological outcomes.

Vitamins have been minimally explored in published kurut literature, with existing studies focusing primarily on minerals or bioactive plant-derived compounds. The broader nutritional enhancement achieved through formulation adjustments in the present work therefore addresses a gap in the field.

Microbiological characteristics show the most striking contrasts. Traditional kurut often demonstrates considerable microbial contamination. Patır & Ateş reported mesophilic counts of 3.4 × 10^4^ CFU/g, lactic acid bacteria and yeasts/molds near 10^4^ CFU/g, and the presence of Escherichia coli and Staphylococcus aureus in 12% and 16% of samples, respectively [36]. Atasever and Atasever similarly detected coliforms and Staphylococcus–Micrococcus groups at measurable levels [37]. İstanbullugil et al. documented total mesophiles at 4.45 log CFU/g and elevated yeast–mold loads, with plant extracts required to partially suppress these groups [40]. The qualitative survey by Khamzaeva confirmed that commercial and homemade kurut commonly contain abundant lactic acid bacteria, including Lactobacillus bulgaricus, Lactococcus lactis and other cocci, but without quantification [43]. None of these studies demonstrated compliance with modern microbiological safety standards. In contrast, the current formulations exhibited substantially reduced total viable counts (≤1.2 × 10^3^ CFU/g), yeast–mold levels ≤20 CFU/g, and complete absence of coliforms, *Enterobacteriaceae*, *E. coli*, *S. aureus*, *Salmonella* and *Listeria monocytogenes*, reflecting the combined influence of controlled fermentation, low water activity, and optimized ingredient ratios.

Sensory profiling revealed a clear formulation trade-off between nutritional modification and preservation of the traditional sensory profile. Variants with higher levels of plant and concentrated protein ingredients tended to show intensified sourness, darker appearance, or firmer, less typical texture, which reduced overall liking despite improved compositional value. In contrast, variant E2 achieved the most favorable balance: it maintained the characteristic fermented-milk flavor as well as familiar color and mouthfeel while still providing meaningful nutritional improvement, indicating that moderate enrichment levels are required for market-viable fortified kurt.

Collectively, comparison with the existing literature demonstrates that controlled formulation offers significant advantages over traditional and drying-focused approaches in terms of nutritional composition, physicochemical stability, and microbiological safety. These findings position enriched kurt as a promising functional dairy product with enhanced reliability and improved dietary value relative to conventionally produced kurut documented in earlier research.

## 5. Conclusions

This study investigated the modification of kurt formulations through the incorporation of dairy- and plant-derived ingredients under laboratory-scale conditions. Formulation changes significantly affected physicochemical, nutritional, and sensory properties. The incorporation of buttermilk protein sediment, spray-dried whey, soy protein concentrate, and flaxseed cake diversified the protein matrix and improved mineral value, particularly calcium, while maintaining acceptable microbial safety, pH, and water activity. However, total protein content in the modified variants did not exceed the control, indicating qualitative protein modification rather than absolute protein enrichment. In parallel, the developed formulations demonstrated a measurable enrichment in carbohydrates, which varied inversely with protein, reflecting increased lactose contribution from whey powder and polysaccharides from plant ingredients and resulting in higher energy density. Sensory evaluation showed that the level of enrichment strongly influenced product acceptability. Variant E2 provided the most balanced profile, preserving characteristic sensory properties, whereas higher inclusion levels led to greater deviations from the typical sensory characteristics of kurt. Further work should address storage stability, nutrient bioavailability, and broader consumer testing to support scale-up and market relevance.

## Figures and Tables

**Table 1:** Formulations of different variants of kurt.

Ingredient	Control	Experiment 1	Experiment 2	Experiment 3
Katyk (concentrated fermented milk base), %	98	58	62	58
Dry curd whey powder, %	0	18	8	22
Buttermilk (dried), %	0	10	18	6
Soy protein concentrate	0	6	8	2
Flaxseed cake (powder), %	0	6	2	10
Table salt, %	2	2	2	2

**Table 2:** Chemical composition of buttermilk and dry whey powder (g/100 g, wet weight basis).

Name	Moisture	Protein	Fat	Carbohydrates	Ash
Buttermilk	61.45 ± 0.89 ^b^	11.51 ± 0.20 ^b^	17.78 ± 0.28 ^b^	7.53 ± 0.15 ^a^	1.73 ± 0.03 ^a^
Dry whey powder	9.47 ± 0.13 ^a^	10.08 ± 0.11 ^a^	0.67 ± 0.01 ^a^	73.92 ± 1.25 ^b^	4.79 ± 0.08 ^b^

^a,b^ Different lowercase letters indicate statistically significant differences within the columns (*p* < 0.05). All values are expressed as g/100 g of product on a wet weight basis (as received).

**Table 3:** Amino acid composition of buttermilk and dry whey powder, g/100 g.

Amino Acid	Buttermilk	Dry Whey Powder
Arginine	1.179 ± 0.014 ^b^	0.613 ± 0.012 ^a^
Lysine	1.220 ± 0.015 ^b^	0.755 ± 0.014 ^a^
Tyrosine	0.895 ± 0.013 ^b^	0.208 ± 0.004 ^a^
Phenylalanine	0.813 ± 0.011 ^b^	0.470 ± 0.010 ^a^
Histidine	0.691 ± 0.005 ^b^	0.525 ± 0.009 ^a^
Leucine + isoleucine	1.342 ± 0.018 ^b^	0.908 ± 0.014 ^a^
Methionine	0.610 ± 0.009 ^b^	0.317 ± 0.006 ^a^
Valine	1.220 ± 0.021 ^b^	0.766 ± 0.013 ^a^
Proline	1.830 ± 0.023 ^b^	1.313 ± 0.017 ^a^
Threonine	1.017 ± 0.015 ^b^	0.832 ± 0.017 ^a^
Serine	1.057 ± 0.015 ^b^	0.646 ± 0.010 ^a^
Alanine	0.651 ± 0.008 ^b^	0.525 ± 0.013 ^a^
Glycine	0.447 ± 0.005 ^b^	0.219 ± 0.003 ^a^

^a,b^ Different lowercase letters indicate statistically significant differences within the rows (*p* < 0.05).

**Table 4:** Chemical composition of katyk.

Indicator	Mean ± SD
Protein, %	18.83 ± 0.35
Fat, %	3.91 ± 0.02
Carbohydrates, %	5.06 ± 0.08
Moisture, %	69.48 ± 1.13
Ash, %	1.42 ± 0.02

**Table 5:** Amino acid composition of katyk.

Amino Acid	Content
Arginine	1.261 ± 0.017
Lysine	1.873 ± 0.030
Tyrosine	1.513 ± 0.020
Phenylalanine	1.045 ± 0.015
Histidine	0.829 ± 0.015
Leucine + isoleucine	1.945 ± 0.027
Methionine	0.865 ± 0.015
Valine	1.657 ± 0.020
Proline	3.026 ± 0.049
Threonine	1.153 ± 0.019
Serine	1.441 ± 0.029
Alanine	0.829 ± 0.016
Glycine	0.504 ± 0.008

**Table 6:** Vitamin and mineral composition of katyk, mg/100 g.

Vitamin/Mineral	Content
Vitamin A	0.046 ± 0.001
Vitamin E	0.220 ± 0.003
Vitamin C	0.380 ± 0.003
Ca	309.4 ± 6.9
Mg	27.81 ± 0.26

**Table 7:** Chemical composition of kurt.

Variant	Water	Ash	Protein	Fat	Carbohydrates
Control	13.61 ± 0.18 ^b^	9.56 ± 0.13 ^b^	52.04 ± 0.66 ^d^	10.8 ± 0.19 ^c^	13.99 ± 0.18 ^a^
Experiment 1	11.68 ± 0.17 ^a^	7.95 ± 0.12 ^a^	34.41 ± 0.46 ^b^	8.59 ± 0.17 ^b^	37.37 ± 0.51 ^c^
Experiment 2	11.83 ± 0.19 ^a^	8.24 ± 0.14 ^a^	40.99 ± 0.64 ^c^	12.05 ± 0.24 ^d^	26.89 ± 0.50 ^b^
Experiment 3	11.72 ± 0.16 ^a^	7.96 ± 0.09 ^a^	30.67 ± 0.39 ^a^	7.80 ± 0.11 ^a^	41.85 ± 0.65 ^d^

^a–^^d^ Different lowercase letters indicate statistically significant differences within the columns (*p* < 0.05).

**Table 8:** Amino acid composition of kurt, %.

Amino Acid	Control	Experiment-1	Experiment-2	Experiment-3
Lysine	10.44 ± 0.17 ^b^	8.87 ± 0.10 ^a^	8.99 ± 0.12 ^a^	8.76 ± 0.14 ^a^
Leucine	nd	2.67 ± 0.05 ^b^	2.41 ± 0.04 ^a^	2.88 ± 0.05 ^c^
Isoleucine	nd	1.62 ± 0.03 ^b^	1.44 ± 0.03 ^a^	1.72 ± 0.03 ^b^
Leucine + Isoleucine	10.84 ± 0.15 ^d^	8.64 ± 0.14 ^c^	7.45 ± 0.09 ^b^	6.86 ± 0.09 ^a^
Valine	9.24 ± 0.16 ^c^	8.13 ± 0.12 ^b^	7.91 ± 0.11 ^ab^	7.65 ± 0.10 ^a^
Threonine	6.43 ± 0.14 ^b^	6.22 ± 0.13 ^ab^	5.94 ± 0.09 ^a^	5.90 ± 0.07 ^a^
Methionine	4.82 ± 0.07 ^c^	3.37 ± 0.04 ^a^	3.69 ± 0.04 ^b^	3.44 ± 0.06 ^a^
Tryptophan	nd	0.38 ± 0.01 ^a^	0.41 ± 0.01 ^b^	0.49 ± 0.01 ^c^
Phenylalanine	5.82 ± 0.09 ^a^	5.61 ± 0.05 ^a^	5.68 ± 0.10 ^a^	5.61 ± 0.07 ^a^
Tyrosine	8.43 ± 0.13 ^c^	6.84 ± 0.08 ^b^	6.6 ± 0.06 ^b^	6.11 ± 0.08 ^a^
Arginine	7.03 ± 0.12 ^a^	7.21 ± 0.13 ^a^	7.39 ± 0.15 ^a^	7.28 ± 0.09 ^a^
Histidine	4.62 ± 0.10 ^b^	4.16 ± 0.06 ^a^	4.11 ± 0.07 ^a^	4.03 ± 0.05 ^a^
Serine	8.03 ± 0.15 ^c^	7.42 ± 0.12 ^b^	7.22 ± 0.13 ^ab^	7.04 ± 0.09 ^a^
Alanine	4.62 ± 0.06 ^a^	4.52 ± 0.09 ^a^	4.61 ± 0.08 ^a^	4.66 ± 0.06 ^a^
Glycine	2.81 ± 0.03 ^a^	3.25 ± 0.05 ^b^	3.33 ± 0.04 ^b^	3.36 ± 0.05 ^b^
Proline	16.87 ± 0.27 ^b^	13.18 ± 0.22 ^a^	12.94 ± 0.28 ^a^	12.42 ± 0.19 ^a^
Aspartic acid	nd	3.11 ± 0.03 ^a^	3.55 ± 0.07 ^b^	4.24 ± 0.09 ^c^
Glutamic acid	nd	5.47 ± 0.07 ^a^	5.89 ± 0.09 ^b^	7.03 ± 0.14 ^c^
Cysteine	nd	0.48 ± 0.01 ^b^	0.43 ± 0.01 ^a^	0.52 ± 0.01 ^c^

^a–d^ Different lowercase letters indicate statistically significant differences within the rows (*p* < 0.05). nd—not detected.

**Table 9:** Mineral composition of kurt.

Minerals, mg/100 g	Control	Experiment-1	Experiment-2	Experiment-3
Calcium (Ca), mg	750.0 ± 11.2 ^a^	856.0 ± 15.6 ^b^	732.8 ± 7.7 ^a^	880.7 ± 18.2 ^b^
Magnesium (Mg), mg	38.5 ± 0.41 ^a^	85.5 ± 1.15 ^c^	71.1 ± 0.88 ^b^	88.4 ± 1.43 ^c^

^a–c^ Different lowercase letters indicate statistically significant differences within the rows (*p* < 0.05).

**Table 10:** Sensory characteristics of kurt samples.

Indicator	Control	Experiment 1	Experiment 2	Experiment 3
Appearance	Spherical shape	Spherical shape	Spherical shape	Spherical shape
Taste	Sour milk, slightly salty	Sour milk with a slight taste of flaxseed meal and whey, slightly salty	Sour milk, slightly salty	Sour milk with a distinct taste of flaxseed meal and whey, slightly salty
Smell	Sour milk	Sour milk	Sour milk	Sour milk, faint smell of whey
Consistency	Hard, dry	Hard, dry	Hard, dry	Hard, dry, crumbly
Color	Light yellow	Light brown	Light brown	Dark brown

**Table 11:** Microbiological indicators of different variants of kurt samples.

Microbiological Indicators	Permissible Concentration Indicators [35]	Control	Experiment-1	Experiment-2	Experiment-3
Total mesophilic aerobic microbial count, CFU/g	5 × 10^5^	1 × 10^3^	1.2 × 10^3^	9.0 × 10^2^	6.0 × 10^2^
Salmonella, the volume (mass) of product in which not permitted in 25 g	Not permitted	Not detected	Not detected	Not detected	Not detected
Escherichia coli bacteria, volume (mass) of the product in which they are not permitted in 0.1 g	Not permitted	Not detected	Not detected	Not detected	Not detected
Staphylococcus aureus, volume (mass) of product in which not permitted in 1.0 g	Not permitted	Not detected	Not detected	Not detected	Not detected
Listeria monocytogenes volume (mass) of product in which it is not permitted in 25 g	Not permitted	Not detected	Not detected	Not detected	Not detected
Yeast, CFU/g	50	10	15	8	5
Molds, CFU/g	100	10	20	12	7

## Data Availability

The original contributions presented in this study are included in the article. Further inquiries can be directed to the corresponding authors.
